# Supplementary material for: Dysregulated Gut Homeostasis Observed Prior to the Accumulation of the Brain Amyloid-β in Tg2576 Mice
Source: Int J Mol Sci. 2020 Mar 3;21(5):1711. doi: 10.3390/ijms21051711 (PMC7084806; doi:10.3390/ijms21051711)
Supplement: Supplementary file 1 [file ijms-21-01711-s001.pdf]

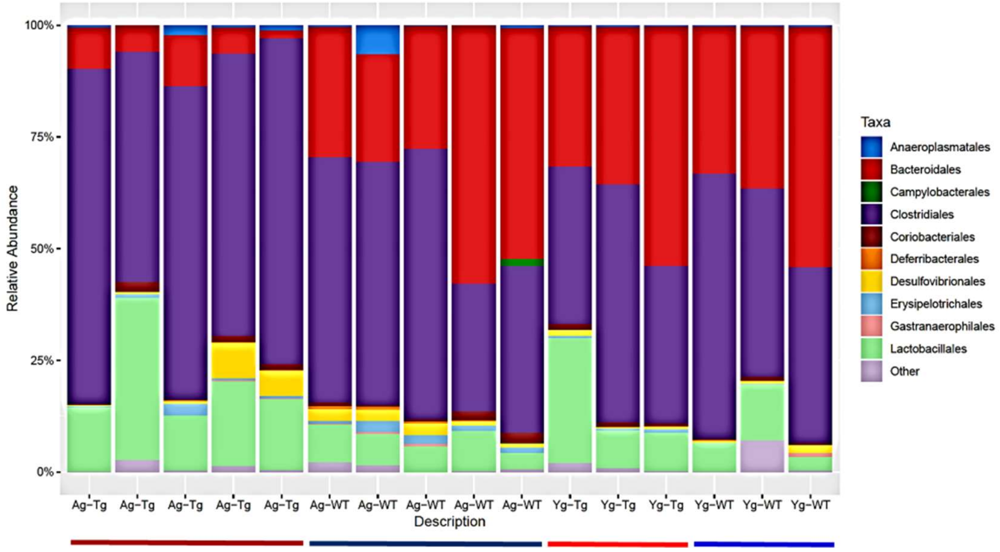

Supplementary Figure S1 – Pre-symptomatic shift in bacterial composition observed in Tg2576 at 6 months (Yg-Tg) compared to WT littermates but was not statistically significant, likely due to small sample size.

Supplementary Table 1 - Post-mortem autopsy samples from AD patients including five brain specimens (hippocampal region) and two corresponding large intestinal autopsies from patients diagnosed with AD. Additionally, gut and brain tissues from control patients who never had AD or any other type of dementia were used as experimental controls.

	Sample ID	Age	Sex	Pathology	Samples	Figure name
**AD**	HA-14-13	69 yrs	Female	AD	Brain & Gut	AD-1
	HA-14-50	77 yrs	Female	AD	Brain & Gut	AD-2
	HA-15-61	75 yrs	Female	AD	Brain	AD-3
	HA-15-67	71 yrs	Female	AD	Brain	AD-4
	HA-18-45	88 yrs	Male	AD	Brain	AD-5
**Control**	HA-18-15	78 yrs	Male	Control	Brain & Gut	Control-1
	HA-18-73	79 yrs	Male	Control	Brain & Gut	Control-2
	HA-18-13	65 yrs	Male	Control	Brain	Control-3
